# Inheritance of Alcohol Abuse: Cross-Fitting Analysis of Adopted Men

**Published:** 1995

**Authors:** Andrew C. Heath

**Affiliations:** Andrew C. Heath, D.Phil., is in the Department of Psychiatry, Washington University School of Medicine, St. Louis, Missouri

**Keywords:** hereditary factor, AOD abuse, adoptive family relation, adoption study, environmental factors, risk factors

**Figure:**
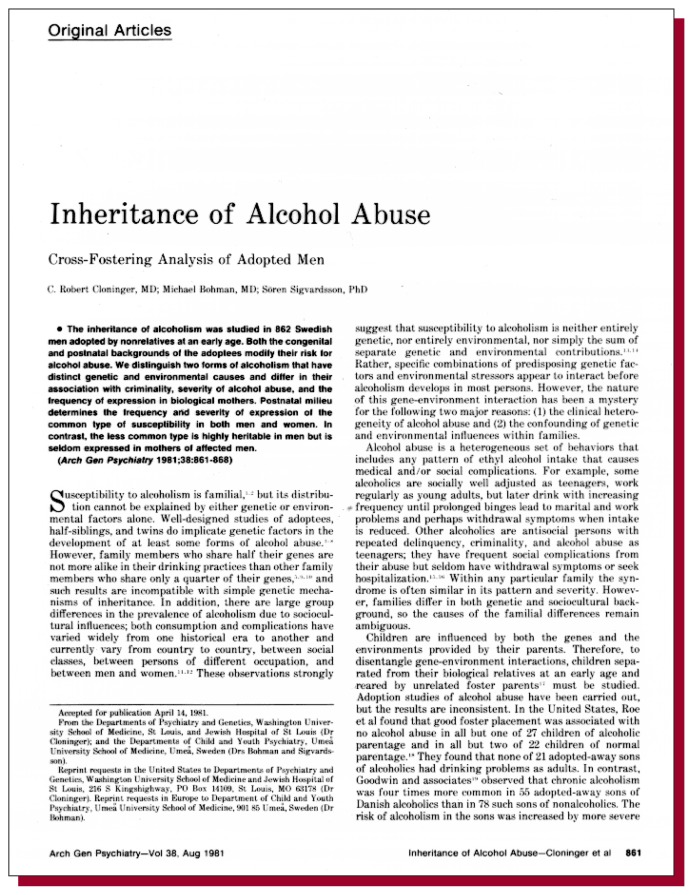
Cloninger, C.R.; Bohman, M.; and Sigvardsson, S. Inheritance of alcohol abuse: Cross-fostering analysis of adopted men. *Archives of General Psychiatry* 38:861–868, 1981.

Of all the articles published on the genetic contribution to alcoholism risk, this 1981 article by Cloninger and colleagues and its companion, “Maternal inheritance of alcohol abuse: Cross-fostering analysis of adopted women,” by Bohman and colleagues, also published in 1981, have perhaps had the greatest impact in this area. Based on analysis of alcohol-related registrations with the Swedish Temperance Board of adoptees and their biological and adoptive parents, Cloninger and colleagues reported finding two subtypes of alcoholism: type II, or “male-limited” alcoholism, which was observed predominantly in males and was strongly influenced by heredity, and type I, or “milieu-limited” alcoholism, which was observed in both men and women and was less strongly influenced by heredity. In type I, postnatal environment was found to have an important moderating effect on the inherited risk of the disease. Thus, in this article, Cloninger and colleagues for the first time sought to address in one study the issues of genetic involvement in the development of alcoholism, gender differences in the inheritance of alcoholism, the existence of subtypes of alcoholism, and the interaction of both genetic makeup (i.e., genotype) and environment in the development of disease (i.e., differences in the importance of genetic risk as a function of environmental exposure). Cloninger further elaborated on this work through an attempt to relate alcoholic subtype to the pattern of alcoholic symptoms (Cloninger 1987).

Cloninger and colleagues’ Swedish adoption study was by no means the first to obtain findings consistent with a genetic contribution to alcoholism risk. The pioneering work in the 1950’s of Kaij (published in 1960) showed that in a series of Swedish twins identified as having alcohol-related problems (once again through registrations with the Swedish Temperance Board), the risk that the other twin also had alcohol-related problems was significantly elevated in identical twins (i.e., monozygotic), compared with fraternal twins (i.e., dizygotic). Such a finding would be predicted if genetic influences were important.

This finding was replicated and extended in a 1981 study by Hrubec and Omenn, which offers what is arguably the strongest evidence for a genetic involvement in severe alcoholism. This study was based on a review of U.S. Veterans Administration records of 13,486 male twins identified through their service in World War II. The first large-sample adoption study was the Danish adoption study of Goodwin and colleagues (1974, 1977). They found a significantly elevated alcoholism risk in the adopted-away sons, but not daughters, of biological parents who had been hospitalized with an alcoholism diagnosis, compared with other adoptees who served as controls. Nonetheless, because of the magnitude of the sample (862 male and 913 female adoptees), the study by Cloninger and colleagues, which found a significant association between alcohol problems in adopted children and alcohol problems in the biological but not adoptive parents, provides the most powerful evidence from any adoption study for a genetic contribution to alcoholism risk.

Fourteen years later, the question of whether genetic differences can lead to differences in alcoholism risk is no longer controversial. The most telling evidence comes from Asian samples: A series of studies have found lower frequency of an inherited single-gene deficiency of alcoholism metabolism (i.e., aldehyde dehydrogenase [ALDH2]) deficiency) among alcoholics compared with nonalcoholics (Thomasson et al. 1993).

As has been reviewed elsewhere (Heath et al. in press), the question of whether the genetic contribution to risk of developing alcoholism is greater in women than in men remains more controversial. The theory that there is a gender difference in the prevalence of alcoholism is perfectly compatible with the hypothesis that genes play an equally important role in alcoholism in both genders but that women have a much higher threshold of risk for developing the disease than do men. Fortunately, data on Swedish Temperance Board registrations for the adoptees and their biological parents (excluding information about alcohol-related hospitalizations and other information available on the adoptees) have been published in a form that permits reanalysis. These data, however, do not reveal a significant gender difference in the heritability of alcoholism.

Data from the Virginia twin study (Kendler et al. 1992), an interview survey of a general population sample of more than 1,000 female twin pairs, are consistent with a genetic contribution to alcohol dependence in women that accounts for 55 percent of the variation in risk. This figure is very close to the 63-percent heritability that is estimated from the U.S. male twin data of Hrubec and Omenn (1981). Even data from studies using clinically ascertained alcoholics and their relatives (e.g., the Goodwin data [1977] or the Minnesota twin studies of Pickens and colleagues [1991] and McGue and colleagues [1992]) when reanalyzed using appropriate statistical techniques are consistent with equal heritability of alcoholism in men and women (Heath et al. in press).

It is not possible to address fully here the controversy over whether alcoholism is a heterogeneous disorder, with different subtypes of alcoholism having different patterns of inheritance. The analyses of Cloninger and colleagues reviewed in this article, like all such analyses, need replication in an independent sample. Subsequent attempts to subtype alcoholics on the basis of alcoholic symptom profile or personality type have not been overwhelmingly successful. Perhaps there are many different pathways to alcoholism and the expression of alcoholic symptoms is largely determined by the severity of alcoholism rather than by subtype.

In closing, perhaps the single most important issue raised by Cloninger and colleagues was that by studying the interaction of genes and the environment, researchers will be better able to identify ameliorating environments for those at high genetic risk of developing alcoholism. Although their statistical analyses on this topic are still debatable, the emphasis on this concept was an important one. With the exception of Cadoret and colleagues’ adoption studies (1985, 1987), this issue has not yet received, from those studying the genetic contribution to risk of psychiatric disorder, the attention that it deserves.
